# Mother knows worst? Fungal infection enhances corn flavonoid of wogonin to inhibit *Conogethes punctiferalis* larval growth

**DOI:** 10.1111/pbi.70051

**Published:** 2025-03-19

**Authors:** Qian Li, Jiayu Li, Kaining Wu, Yue Tong, Aihuan Zhang, Yanli Du

**Affiliations:** ^1^ College of Bioscience and Resource Environment/Key Laboratory of Urban Agriculture (North China), Ministry of Agriculture and Rural Affairs of the People's Republic of China Beijing University of Agriculture Beijing China

**Keywords:** *Conogethes punctiferalis*, *Trichoderma asperellum*, wogonin, gut microbiota, juvenile hormone, growth

## Abstract

Pathogen infection in host plants can alter the attraction and adaptability of herbivorous insects. Female adult insects often exhibit selective behaviours based on their environmental experiences, enabling their offspring to avoid adverse conditions and ensuring healthy growth and development. However, comprehensive studies integrating both the perspectives of offspring fitness and host plant to validate the selective significance of such parental ‘Mother knows worst’ experiences remain limited. Building on our previous findings that female *Conogethes punctiferalis* (Yellow peach moth, YPM) adults exhibit oviposition avoidance behaviour towards corn infected with *Trichoderma asperellum*, we further confirmed that corn infected by *T. asperellum* significantly inhibits the growth and development of YPM larvae. Feeding on infected corn decreases larval gut microbiota diversity, core microbiota abundance and led to differential expression of key genes in juvenile hormone metabolic pathway. Moreover, the content of flavonoid wogonin, a secondary metabolite, was significantly increased in infected corn. In vitro feeding experiments revealed that wogonin negatively impacts YPM larval growth by causing the juvenile hormone accumulation and suppressing the abundance of core gut microbial strains. This study validates the adaptive significance of parental empiricism from the perspective of offspring, while further elucidating the mechanisms by which microbial‐mediated plant resistance against insects, as well as for exploring and utilizing effective biocontrol resources against YPMs.

## Graphical abstract

Schematic representation of *Trichoderma asperellum* infection affects the flavonoid content in corn and influences the gut microbiota and juvenile hormone pathway of *Conogethes punctiferalis* larvae leading to growth inhibition.

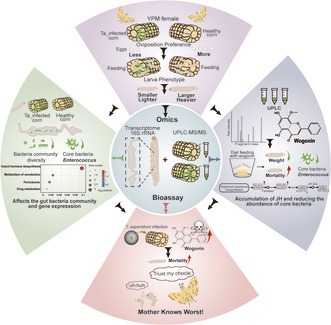

## Introduction

In natural ecosystems, plant pathogens and herbivorous insects frequently coexist on the same host plants, forming intricate interaction networks among host plants, insects and pathogens (Raman and Suryanarayanan, 2017). These pathogens directly affect herbivorous insects by altering plant leaf nutrient composition or colonizing insect guts to provide essential nutrients (Eberl *et al*., 2019, 2020). Indirectly, they modify plant volatile profiles, influencing insect oviposition and feeding behaviours (Cha *et al*., 2020; Gu *et al*., 2022). Consequently, plant pathogens add complexity to plant–insect interactions, driving shifts in herbivore preferences and adaptations.

Behavioural responses shaped by parental environments are widely observed across taxa, ranging from animals to insects, indicates that parents adjust their offspring's life history strategies based on their own environmental experiences (Zhu *et al*., 2024). This adjustment enhances the offspring's ability to avoid adverse conditions and ensures healthy growth and development (Heard and Martienssen, 2014). For instance, brown planthoppers prefer to lay eggs on rice plants damaged by the striped stem borer, exploiting herbivory‐induced plant volatiles to create a predator‐free space for their progeny (Hu *et al*., 2020; Jiao *et al*., 2018). Similarly, solitary locusts produce more, smaller and irregularly shaped eggs, while gregarious locusts lay fewer, larger and more synchronized eggs, facilitating early adaptation to environmental challenges and shaping population dynamics (Chen *et al*., 2015; He *et al*., 2022). These maternal strategies‐spanning reproduction, oviposition and other behaviours‐consistently enhance offspring survival and increase genetic fitness, encapsulated in the ‘Mother knows best’ principle (Kohandani *et al*., 2017).

Within the dynamic interactions among pathogens, insects and plants, an evolutionary arms race has developed. Parental insects exhibit selective behavioural responses to host plants influenced by pathogenic microorganisms. For example, *Candidatus liberibacter asiaticus* infection in citrus trees reduces the release of volatiles that attract natural enemies, favouring the feeding and oviposition preferences of psyllids *Diaphorina citri* (Lin *et al*., 2016). Additionally, the yeast pathogen *Saccharomyces cerevisiae* emits volatiles that induce feeding and oviposition preferences in female fruit flies (Becher *et al*., 2012). Volatile compounds released by *Penicillium* also attract oriental fruit flies for oviposition, with the fungal entering the fly gut to supply pyridoxine, enhancing emergence rates and shortening developmental time (Gu *et al*., 2022). Our previously studies showed that female YPM exhibit oviposition preferences for apples infected with *Penicillium* (Guo *et al*., 2022). Feeding on fungi‐infected apples enhances larval digestive capacity and overall fitness (Li *et al*., 2024). These findings further support the applicability of the ‘Mother Knows Best’ principle within the different insects. The intricate interactions are likely shaped by factors such as microbially induced plant volatile emissions and changes in metabolites, determining whether the behavioural responses of parental insects are beneficial or detrimental in this microbe–plant symbiotic relationship (Franco *et al*., 2021; Keesey *et al*., 2017). Currently, most studies focus on parental behavioural strategies that favour offspring growth. However, pathogenic infections can also trigger plants to repel herbivorous insects (Stensmyr *et al*., 2012), leading to a reduced oviposition rate – a phenomenon that may be described as the ‘Mother Knows Worst’ principle. Nevertheless, there is still a lack of comprehensive evidence from the perspectives of both offspring and host plants to validate the correctness of parental behavioural preferences shaped by environmental experiences.

The yellow peach moth (*Conogethes punctiferalis*, YPM), a destructive pest known for its larval boring behaviour, infests a wide range of field and economic crops (Li *et al*., 2024). In recent years, the extensive cultivation of summer corn in China, the increased planting of economic crops and global warming have intensified the threat posed by YPM to corn. This is often exacerbated by synergistic diseases, leading to significant declines in both corn quality and yield. Notably, YPM demonstrates resilience against pathogen infections in the field and thrives under complex and adverse environmental conditions, making the pathogen‐YPM‐corn interaction a valuable model for studying microorganism‐insect‐plant dynamics. In our preliminary study, we isolated a pathogenic strain of *Trichoderma asperellum* from maize ear rot. Behavioural assays indicated that YPM adults exhibit strong repellence to corn infected with *T. asperellum*, and mated females employ oviposition avoidance strategies (Chen *et al*., 2023). However, aside from the influence of host plant volatiles, it remains unclear whether the oviposition avoidance behaviour of female YPMs is connected to the effects of infected corn on their offspring's growth and development. Specifically, the potential impacts and underlying mechanisms of such parental ‘Mother Knows Worst’ experiences at the offspring level are yet to be elucidated.

In this study, we found that *T. asperellum*‐infected corn significantly inhibits YPM larvae growth and development. Feeding on infected corn alters the diversity and abundance of gut microbiota in larvae, while inducing the differential expression of hormone‐related genes. Additionally, the content of flavonoid secondary metabolite wogonin in infected corn significantly increases. Through in vitro bioassays, we demonstrated that wogonin causes accumulation of larval juvenile hormone and suppresses the abundance of core gut bacteria, ultimately impairing larval growth.

## Results

### *Trichoderma asperellum* inoculated corn inhibits the growth of YPM larvae

**Figure 1:**
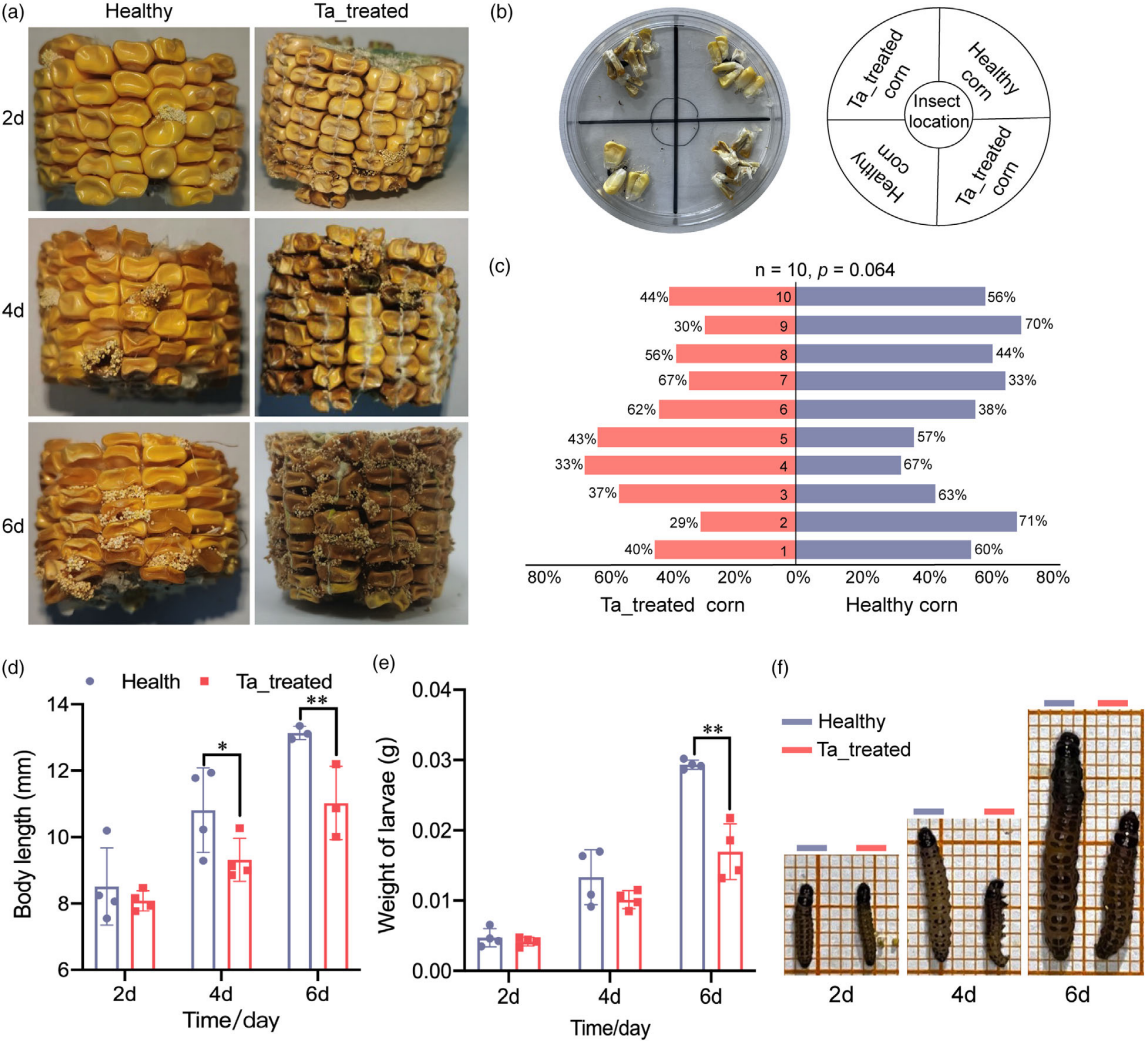
The impact of feeding on corn infected with *Trichoderma asperellum* on the growth of YPM larvae. (a) Phenotypes of corn infected with *T. asperellum* and healthy corn at 2, 4 and 6 days; (b) Selection behaviour test; (c) Selection behaviour of YPM larvae in response to infected versus healthy corn; (d–f) Effects of feeding on infected and healthy corn on the body length, weight and phenotype of YPM larvae.

We investigated the potential effects of *T. asperellum* inoculated corn on the growth and development of YPM larvae by measuring their body length and weight after feeding on the infected corn for 2, 4 and 6 days (Figure 1a). To eliminate any possibility of non‐selective feeding behaviour towards the infected corn, we conducted preference tests, which indicated no significant feeding preference between the infected and healthy maize (Figure 1b,c). Subsequently, we observed that continuous feeding on the Ta‐treated corn for 4 and 6 days significantly inhibited larval body length compared to those fed healthy corn. Additionally, after 6 days of continuous feeding, the larvae exhibited a marked decrease in body weight (Figure 1d,e). Phenotypic observations confirmed that larvae feeding on the infected corn were notably smaller in size (Figure 1f).

### Variations in gut bacterial composition and structure of YPM larvae feeding on healthy versus *T. asperellum* inoculated corn

**Figure 2:**
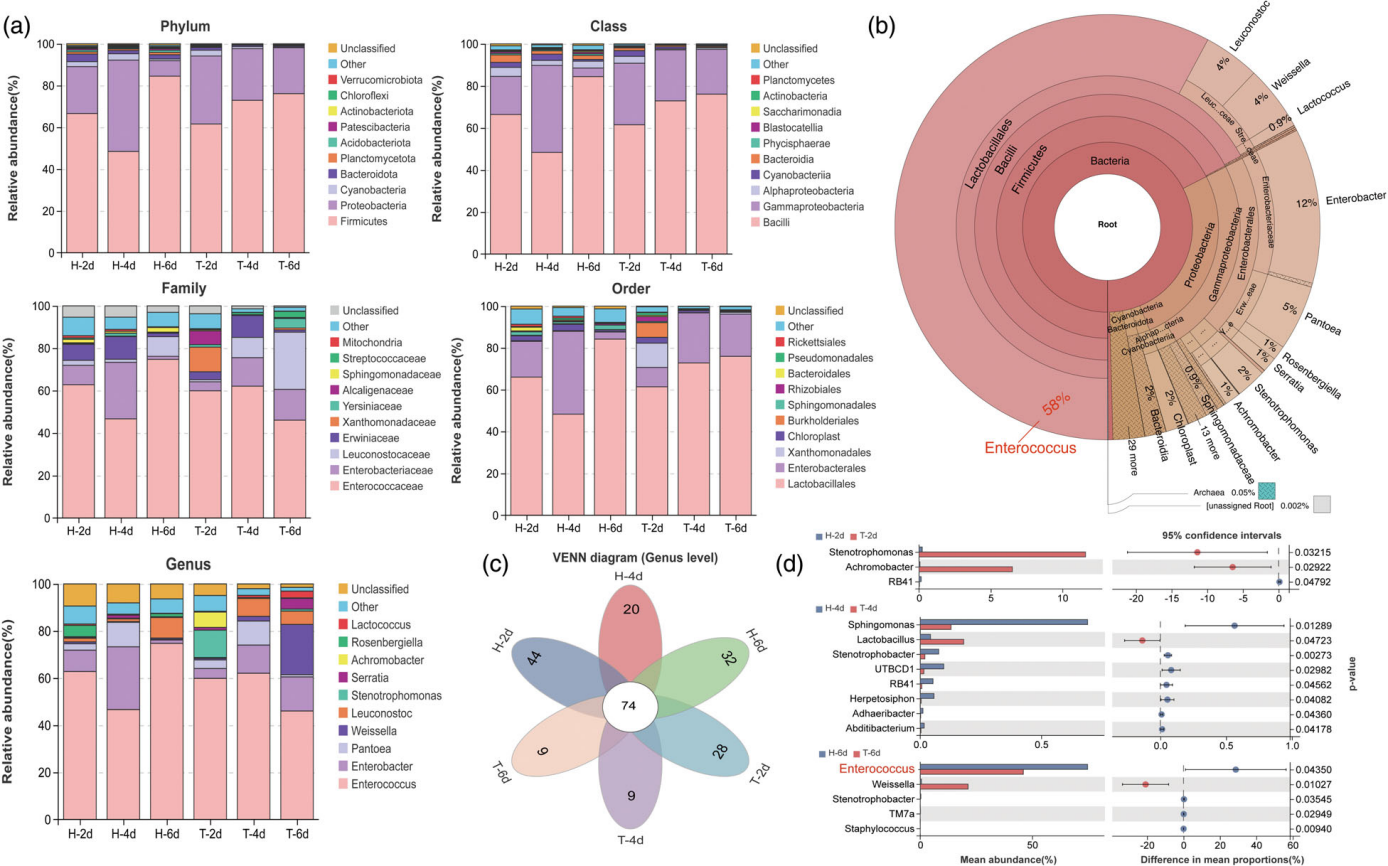
Impact of feeding on *T. asperellum*‐infected corn on the gut bacterial composition and abundance in YPM larvae. (a) The top ten bacterial phylum, class, order, family and genus represented across different treatment groups; (b) The Venn graph based on the 16S rRNA OTUs of different groups; (c) Overall bacterial community composition across all samples; (d) Bacterial taxa with significant differences in relative abundance between larvae fed on infected versus healthy corn over various time periods.

To elucidate whether the phenotypic differences resulting from feeding on Ta‐treated corn are associated with the gut microbiota of YPM larvae, we conducted a comparative analysis utilizing 16S rRNA amplicon sequencing. We assessed the differences in gut microbial communities after feeding on healthy corn and Ta‐treated corn for 2, 4 and 6 days. The findings revealed that the gut microbiota of YPM larvae consuming corn primarily consisted of bacteria from the phyla Firmicutes and Proteobacteria (Figure 2a). Within the Firmicutes, the Lactobacillales order, particularly the Enterococcaceae family represented by the genus *Enterococcus*, was predominant, accounting for 60% of the total community composition across all samples (Figure 2b). Moreover, a Venn diagram at the genus level indicated that a total of 74 bacterial species were shared across different sample treatments. However, compared to larvae feeding on healthy corn, the unique bacterial species identified in the guts of those feeding on fungal‐inoculated corn after 2, 4 and 6 days were 28, 9 and 9, respectively, which were significantly lower than the 44, 20 and 32 found in those consuming healthy corn (Figure 2c). Furthermore, a comparative analysis at the genus level indicated that after 6 days of feeding on fungal‐inoculated corn, the abundance of *Enterococcus* in the gut of YPM larvae was significantly reduced compared to those feeding on healthy corn (Figure 2d).

**Figure 3:**
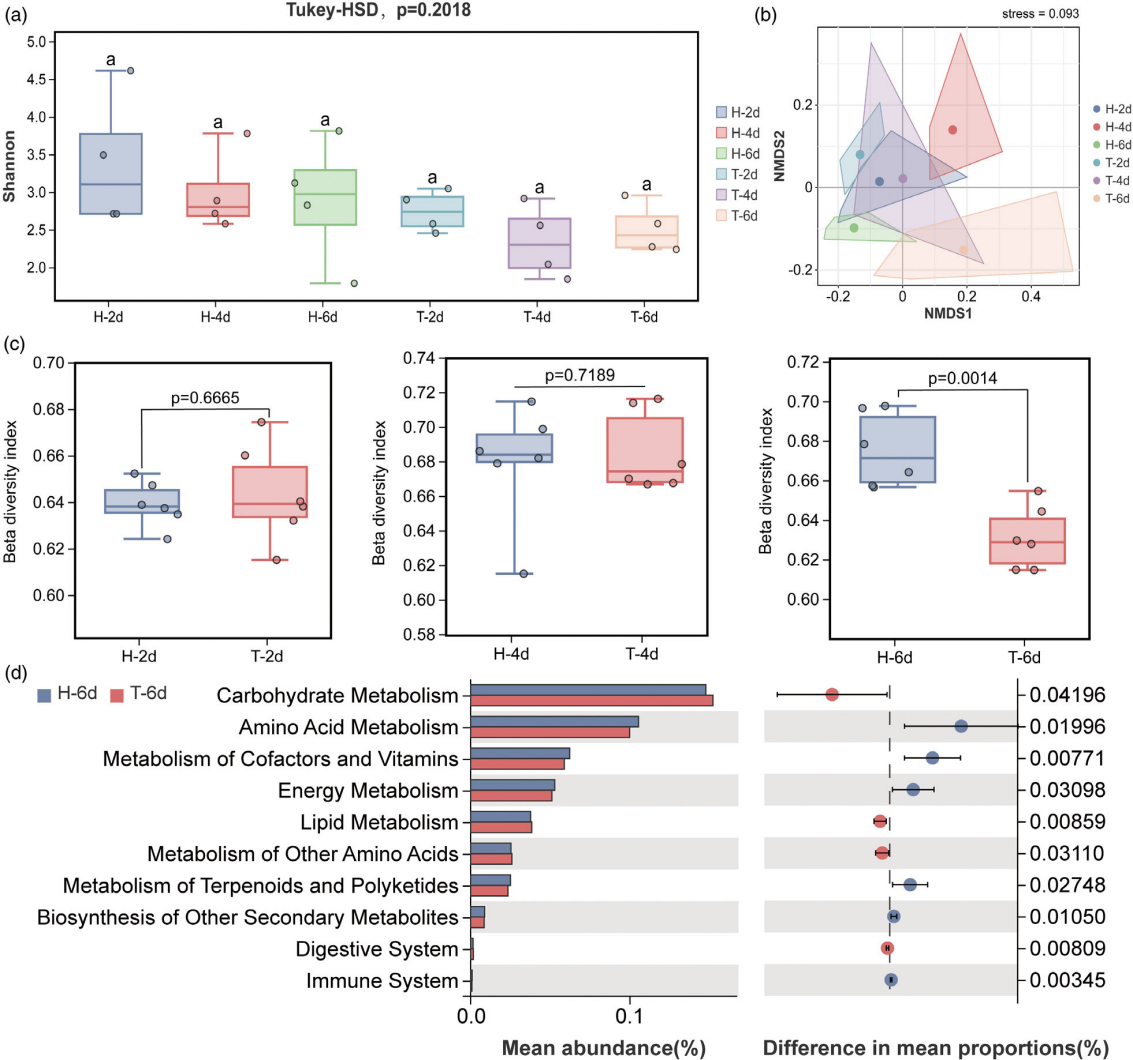
Impact of feeding on *T. asperellum*‐infected corn on the gut bacterial diversity and function in YPM larvae. (a) Impact of feeding on infected corn on the alpha diversity index of YPM larvae; (b) Non‐metric multidimensional scaling (NMDS) plot constructed with Bray–Curtis based on the distribution of OTUs of bacteria across different groups; (c) Analysis of gut bacterial beta diversity in larvae fed on *T. asperellum*‐infected versus healthy corn at 2, 4 and 6 days; (d) Analysis of the functional differences in gut bacteria after 6 days of feeding on *T. asperellum*‐infected versus healthy corn.

Alpha diversity analyses indicated a decreasing trend in the Shannon index of alpha diversity for larvae fed on inoculated corn at different time points compared to those fed on healthy corn; however, the difference was not statistically significant (Figure 3a). To evaluate clustering patterns between Ta‐treated and healthy samples, NMDS analysis based on Bray–Curtis distances was performed. The results showed that samples from larvae fed on healthy corn and Ta‐treated corn for 2 and 4 days were closely clustered together, whereas samples from larvae fed on Ta‐treated corn for 6 days exhibited significant dissimilarity from that fed on healthy corn (Figure 3b), indicating a greater difference in bacterial diversity between treatments on the sixth day. Additionally, no significant difference in the beta diversity index of the gut microbiota of larvae fed on healthy corn and Ta‐treated corn for 2 and 4 days. However, a significant difference was observed on the sixth day (Figure 3c). Moreover, KEGG functional analysis revealed that on the sixth day of feeding on healthy and Ta‐treated corn, distinct microbial communities were primarily engaged in crucial nutrient synthesis pathways, such as carbohydrate metabolism, amino acid metabolism, coenzyme factors and vitamin metabolism (Figure 3d).

### Comparative analysis of the gut transcriptomes of YPM larvae feeding on healthy and *T. asperellum* inoculated corn

**Figure 4:**
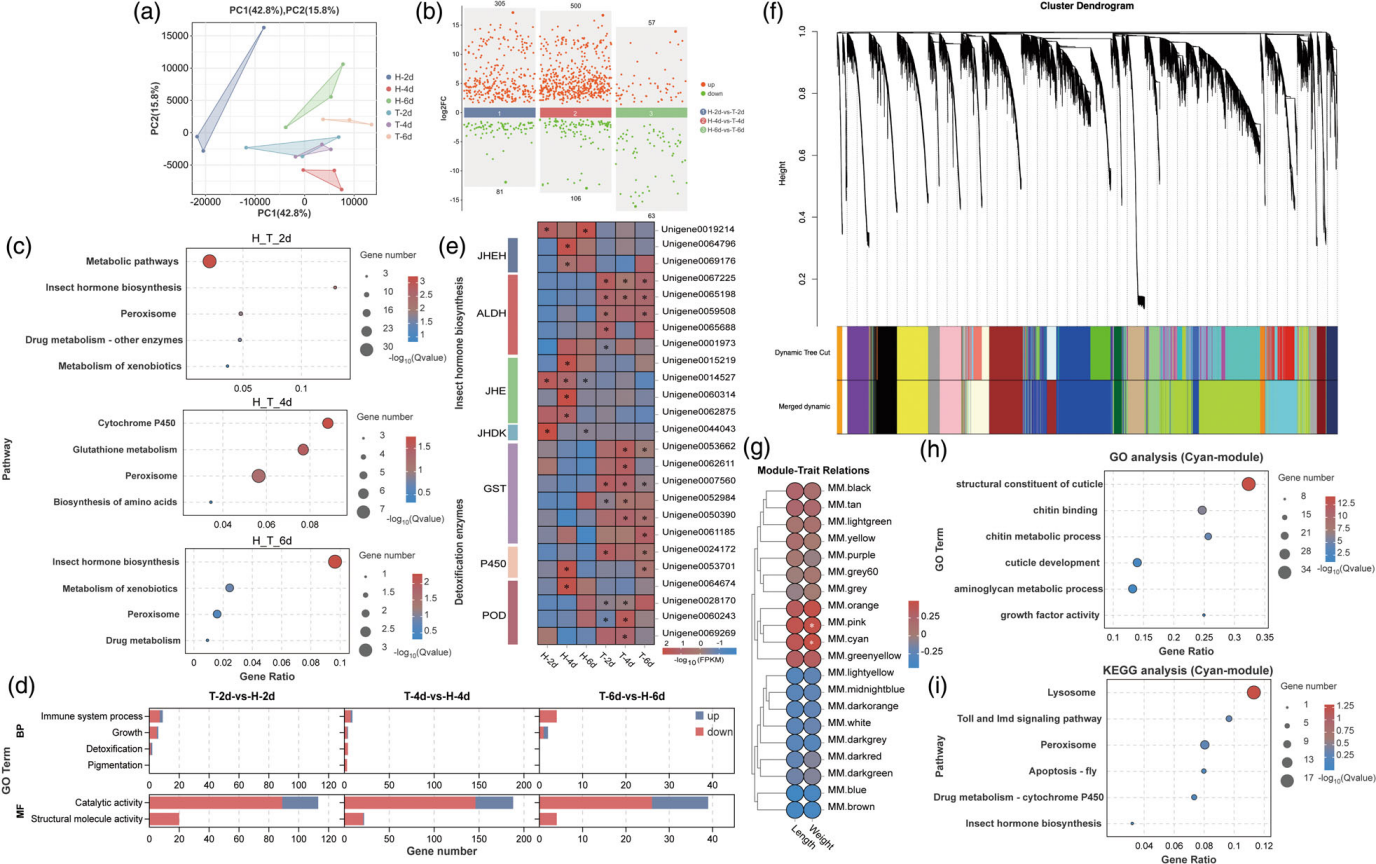
Impact of feeding on *T. asperellum*‐infected corn on the gut gene expression in YPM larvae. (a) PCA analysis of different treatment samples; (b) Number of differentially expressed genes across various time points; (c, d) KEGG and GO functional analysis of differentially expressed genes in larvae fed on *T. asperellum*‐infected versus healthy corn at 2, 4 and 6 days; (e) Differential expression of key genes involved in juvenile hormone synthesis and detoxification pathways in larvae fed on infected versus healthy corn at 2, 4 and 6 days; (f) Module classification of gene expression patterns; (g) Heatmap illustrating the correlation between phenotypic traits and gene modules; (h, i) GO and KEGG functional analysis of gene sets associated with identified modules.

We investigated the differential expression of gut genes in YPM larvae feeding on healthy versus Ta‐treated corn at various time points through transcriptome sequencing. Principal component analysis (PCA) revealed that Ta‐treated corn markedly influences the expression of gut genes in YPM larvae (Figure 4a,b). KEGG pathway analysis of the differentially expressed genes indicated significant enrichment in metabolic pathways, detoxification enzyme activity and insect pheromone synthesis pathways. Additionally, Gene Ontology (GO) annotation highlighted significant enrichment in hydrolysis, immune system functions and structural molecular activity (Figure 4c,d).

Notably, the critical upstream gene in the insect juvenile hormone pathway, aldehyde dehydrogenase (*ALDH*), which converts farnesal into the juvenile hormone precursor farnesoic acid, was significantly upregulated at all time points in larvae fed on Ta‐treated corn. Conversely, key downstream hydrolytic pathway genes, including juvenile hormone epoxide hydrolase (*JHEK*), juvenile hormone esterase (*JHE*) and juvenile hormone diol kinase (*JHDK*), exhibited significantly lower expression levels compared to the control group. Detoxification‐related genes, such as glutathione transferase (*GST*), cytochrome P450 (*P450*) and peroxidase (*POD*), were also markedly upregulated (Figure 4e). Enzyme activity assays confirmed that POD and GST activities significantly increased on days 4 and 6 after feeding on Ta‐treated corn (Figure S1).

To explore gene–phenotype associations, Weighted Gene Co‐Expression Network Analysis (WGCNA) on transcriptome and phenotypic data. Genes with differing expression patterns were categorized into colour‐coded modules (Figure 4f). A heatmap of Pearson correlation coefficients between module Eigen genes and phenotypic traits showed significant correlations between larval weight and the pink (*R* = 0.479, *P* = 0.04) and cyan modules (*R* = 0.499, *P* = 0.035) (Figure 4g). While these modules did not exhibit significant correlations with larval body length, relatively high coefficients were observed (pink: *R* = 0.440, *P* = 0.068; cyan: *R* = 0.406, *P* = 0.095). GO analysis of the gene sets in these modules revealed significant enrichment in pathways related to epidermal structure, immune response and hormone synthesis (Figure 4h,i). The pink module was particularly enriched in pathways related to DNA replication, steroid synthesis and terpenoid synthesis (Figure S2). These findings suggest that phenotypic changes in YPM larvae fed on Ta‐treated corn may be driven by key physiological processes, including hormone synthesis.

### Differential changes in secondary metabolites of Ta‐treated corn revealed by metabolomics

**Figure 5:**
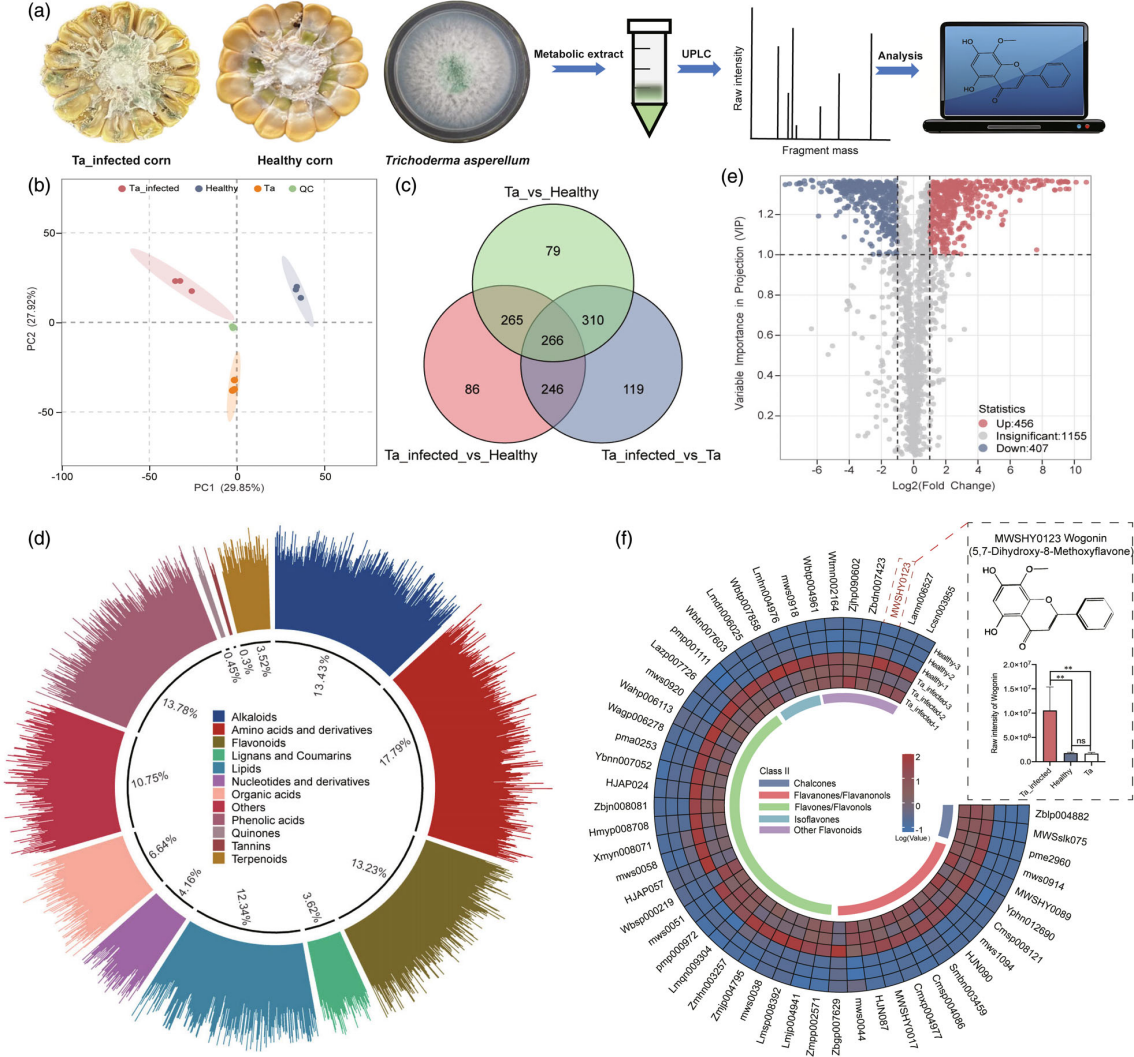
Increased relative content of flavonoid in corn due to *T. asperellum* Infection. (a) Flowchart of the metabolomics analysis process for different samples; (b) PCA analysis of different samples; (c) Venn diagram illustrating differential metabolites among various comparison groups; (d) Pie chart depicting the proportion of primary classifications of metabolites; (e) Volcano plot of differential metabolites after 6 days of feeding on *T. asperellum*‐infected versus healthy corn; (f) Heatmap of differential flavonoid metabolite relative content in different sample and the relative content comparation of wogonin.

To further elucidate the potential factors influencing YPM larval growth, we conducted non‐targeted metabolomic analyses on corn inoculated for 6 days, healthy corn and *T. asperellum* spore suspension (Figure 5a). PCA analysis indicates a high degree of reproducibility within groups while also highlighting notable differences among three treatments (Figure 5b). Additionally, Venn diagram analysis demonstrated substantial disparities among the various comparative groups (Figure 5c). Functional analysis revealed that the differential metabolites encompassed primary metabolites such as amino acids and their derivatives, lipids and nucleotides, as well as secondary metabolites including alkaloids, flavonoids, terpenes, quinones and phenolic acids. Notably, primary metabolites, predominantly represented by amino acids and their derivatives, exhibited significant variations, accounting for 17.39% of the total. In contrast, secondary metabolites were mainly represented by quinones, alkaloids and flavonoids, which constituted 13.78%, 13.43% and 13.23%, respectively (Figure 5d, Table S1).

Additionally, we identified 863 metabolites in the Ta‐treated corn tissues that displayed significant differences when compared to healthy corn. Among these, 456 metabolites were significantly upregulated, while 407 were downregulated (Figures 5e and S3). Functional analysis indicated that the differential expressed metabolites predominantly involved metabolism, plant secondary metabolites biosynthesis, amino acid synthesis and ABC transporter pathways. KEGG enrichment analysis also showed that differential metabolites are mainly enriched in metabolic pathways and amino acid synthesis (Figures S4 and S5). To identify key secondary metabolites that inhibit the growth of YPM larvae, we specifically targeted flavonoid compounds and discovered 51 flavonoids with significantly elevated concentrations. Next, we focused on wogonin, a secondary classification as flavone, characterized by high abundance and the greatest fold change, to further investigate its effects on the growth of YPM larvae in vitro (Figure 5f).

### The negative effects of wogonin on the growth of YPM larvae

**Figure 6:**
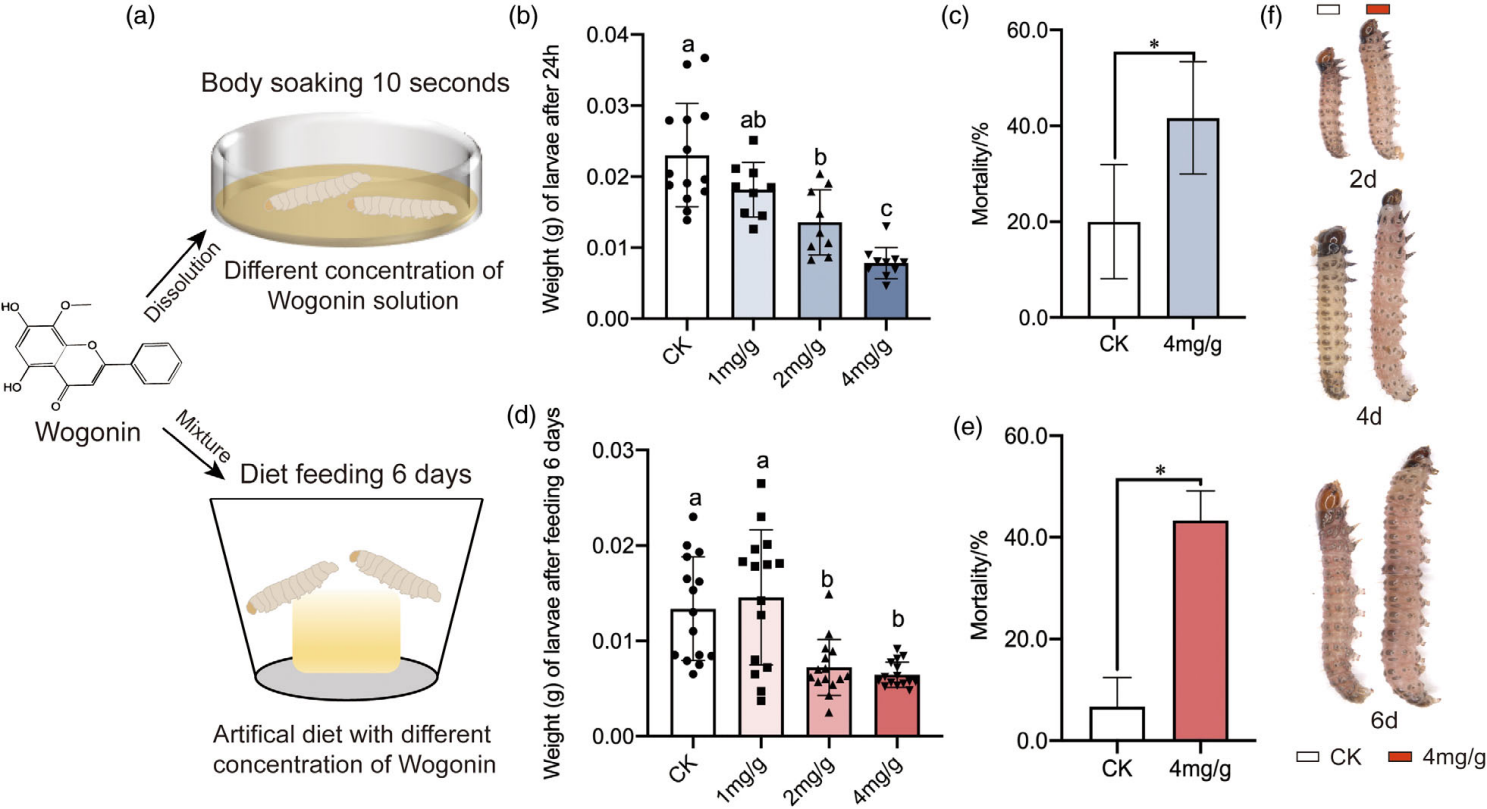
Wogonin soaking and feeding significantly inhibit growth of YPM larvae. (a) YPM larvae soaking in wogonin solution and feeding on wogonin‐contained artificial diet significantly inhibit larval growth; (b) The weight differences of YPM larvae at 24 h after being soaked for 10 s in wogonin solutions at various concentrations; (c) The effect of 4 mg/g wogonin soaking treatment on the mortality rate of YPM larvae; (d) Effects on the weight of YPM larvae after 6 days of feeding on artificial feed containing different concentrations of wogonin; (e) The effect of 4 mg/g wogonin feeding treatment on the mortality rate of YPM larvae; (f) Differences in the phenotype of YPM larvae after feeding on artificial diet containing 4 mg/g of wogonin at different times.

Firstly, we used the method of body wall soaking to immerse the larvae in wogonin solution with different concentration gradients for 10 s, then transferred the larvae to healthy corn and measured the weight of larvae after 24 h (Figure 6a). The results showed that with the increase of wogonin concentration, the weight of YPM larvae showed a decreasing trend. When the concentration reached 2 mg/g and 4 mg/g, the average weight per larva was 0.014 g and 0.008 g, respectively, significantly lower than the weight value of 0.023 g in the control group (*P* < 0.05) (Figure 6b) and the mortality rate also increased significantly (Figure 6c). Furthermore, we also investigated the impact of adding wogonin to artificial diet on the growth of YPM larvae. Similarly, when the concentration of wogonin in artificial diet is 2 mg/g and 4 mg/g, the average weight of YPM larvae after continuous feeding for 6 days was recorded as 0.0072 g and 0.0065 g, respectively, exhibiting a significant decrease compared to the control group with a weight of 0.0123 g (Figure 6d). The mortality rate also exhibited a significant increase (*P* < 0.05) (Figure 6e). Furthermore, phenotypic observations of larvae following varying durations of feeding revealed that those consuming artificial diet containing 4 mg/g wogonin for 2, 4 and 6 days displayed markedly reduced body sizes compared to the control treatment (Figure 6f).

### Wogonin induced differential expression of juvenile hormone metabolic pathway genes and reduction of *Enterococcus* abundance in the core gut microbiota of YPM

**Figure 7:**
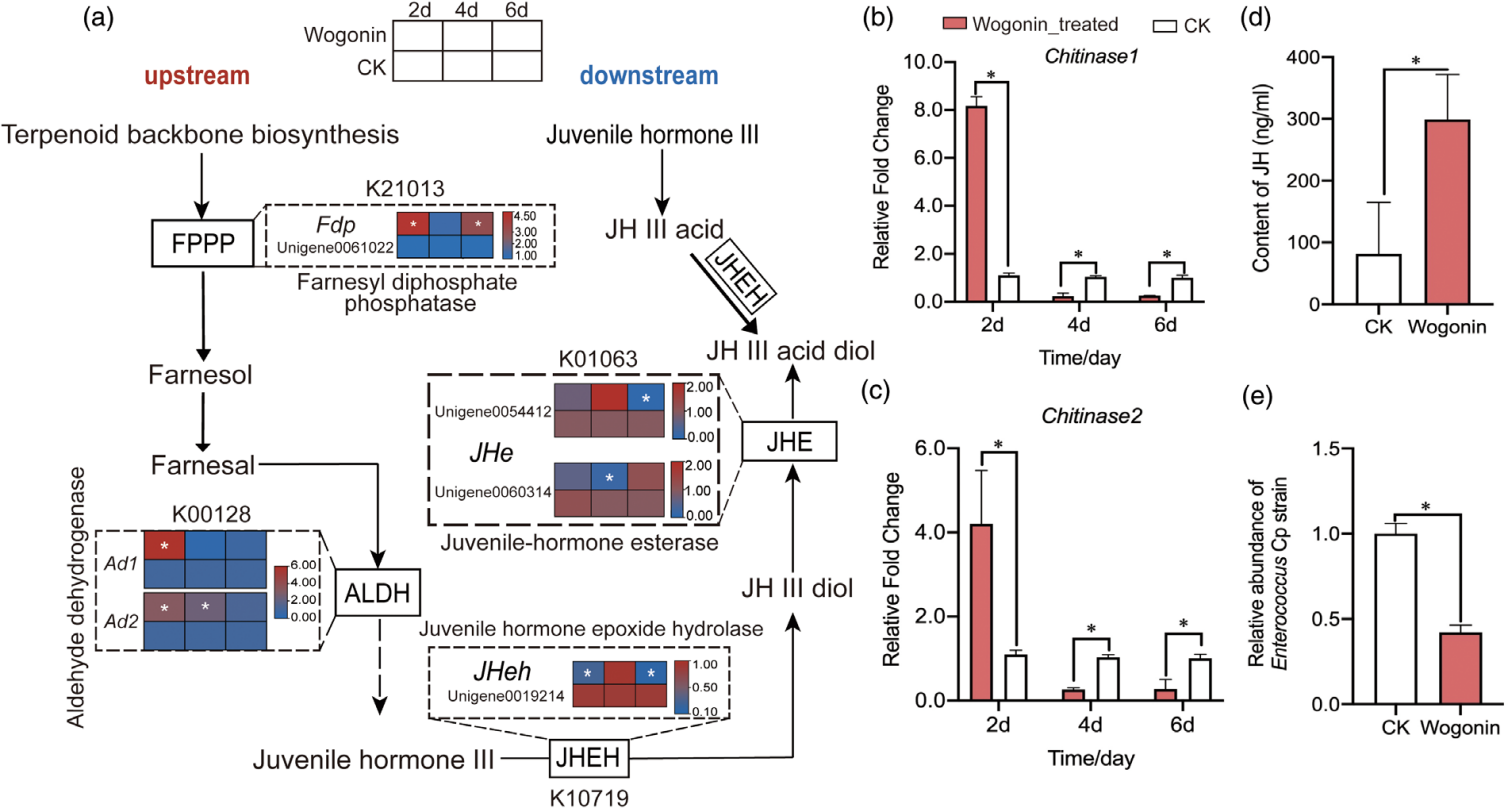
Effects of feeding artificial diets containing wogonin on juvenile hormone pathway gene expression, chitin synthesis genes and core gut microbiota abundance of YPM larvae. (a) Differential expression of key genes in the juvenile hormone pathway; (b, c) Differential expression of chitin synthesis‐related genes; (d) Juvenile hormone content in YPM larvae after 6 days of feeding on artificial diets containing wogonin; (e) Differences in relative abundance of core gut *Enterococcus* in YPM larvae after 6 days of feeding on artificial diets containing wogonin.

Based on previous gut transcriptome data, we further assessed the impact of feeding artificial diet containing 4 mg/g wogonin at different time points using qPCR. The results indicated that, compared to the control group, consumption of the wogonin‐enriched diet significantly increased the expression of the farnesyl diphosphate phosphatase gene (*Fdp*: Unigene0061022) involved in the upstream synthetic pathway of juvenile hormone after continuous feeding for 2 and 6 days. Similarly, key genes in this synthetic pathway, aldehyde dehydrogenase genes (*Ad1*: Unigene0067225, *Ad2*: Unigene0059508), also exhibited significant upregulation after 2 and 4 days of feeding. Conversely, the expression levels of critical downstream degradation pathway genes, juvenile hormone epoxide hydrolase (*JHeh*: Unigene0019214) was significantly downregulated after 2 and 6 days of continuous feeding with the wogonin diet, and the expression levels of esterase genes (*JHe*: Unigene0054412 and Unigene00660314) were significantly downregulated after 6 days and 4 days of continuous feeding, respectively (Figure 7a).

Moreover, we observed a significant upregulation of two chitinase genes (*Chitinase1*: Unigene0061022, *Chitinase2*: Unigene0064025) after 2 days of feeding on the baicalin diet, followed by a marked decrease in expression by the 4th and 6th days (Figure 7b,c). Notably, after 6 days of feeding, the juvenile hormone levels within the larvae significantly increased. It is important to highlight that the abundance of *Enterococcus* Cp strain, a core gut microbiota component, exhibited a significant decline after 6 days of continuous feeding with the wogonin‐enriched diet (Figure 7e).

## Discussion

Building on previous finding that adult female YPMs exhibit oviposition avoidance behaviour towards corn infected with *T. asperellum*, resulting in a reduced egg‐laying quantity (Chen *et al*., 2023), this study further explores the inhibitory effects of *T. asperellum* on YPM larval growth. To investigate the underlying mechanisms, a comprehensive analysis was conducted from both the insect and host plant perspectives. 16S rRNA sequencing revealed distinct changes in the gut microbial community structure and abundance in larvae feeding on Ta‐treated corn compared to those feeding on healthy corn. Specifically, the number of specific bacterial strains decreased significantly at the phylum level, and by the sixth consecutive day of feeding, the abundance of the dominant bacterium *Enterococcus* in the gut decreased significantly while minor microbial populations increased. Gut microbiota play a crucial role in regulating host insect growth, development and environmental adaptability (Gu *et al*., 2022). For instance, bacteria such as *Enterococcus* and *Pseudomonas* in lepidopterans may serve as core members of the gut microbiota, establishing a symbiotic relationship with the host through adaptation to the gut environment, biofilm formation and signal transduction system regulation (Shao *et al*., 2024). In our previous work, we successfully isolated a strain of *Enterococcus mundtii* from the gut of peach borer larvae. Through supplementation experiments, we demonstrated that this strain promotes the growth of YPM larvae primarily by enhancing the activity of digestive enzymes and increasing total protein content in the host (Li *et al*., 2024). Similarly, gut‐associated *Enterococcus* in *Helicoverpa armigera* has been found to alleviate oxidative stress via peroxidase activity (Mazumdar *et al*., 2021). Therefore, the decreased abundance of core bacterial strains may contribute to the observed larval growth inhibition. Environmental and genetic factors, including diet, feeding habits and lineage, strongly influence gut microbiota structure and composition (Juottonen *et al*., 2022). In this study, a significant decrease in the β‐diversity of gut microbiota was observed in larval after 6 days of feeding on Ta‐infected corn, suggesting that dietary differences drive these microbial changes. Comparable findings in *Plagiodera versicolora* show that gut microbial diversity and abundance are linked to feeding preferences and enhanced ecological adaptability (Ma *et al*., 2024). Further research is needed to investigate whether these shifts in microbial community structure and diversity directly impact YPM larval growth.

Through a comparative analysis of the transcriptomic data from YPM larvae guts, we discovered significant differential gene expression in response to feeding on Ta‐treated corn compared to healthy corn. The functions of the differentially expressed genes were predominantly enriched in detoxification metabolism, including P450 genes, peroxidase genes and glutathione S‐transferase genes, all of which were significantly upregulated in the guts of larvae feeding on infected corn. Notably, key genes in the juvenile hormone biosynthesis pathway were upregulated after feeding on infected corn, while crucial genes in the downstream hydrolysis pathway were downregulated. Additionally, WGCNA analysis indicated a significant positive correlation between cuticle formation, immune‐related gene sets and phenotypic changes. The evolution of adaptive traits, including resistance to toxic chemical pesticides (Gould *et al*., 2018) and defence mechanisms against pathogenic microorganisms (Gao *et al*., 2021), often comes with fitness costs. For instance, in aphids, p38 and ERK kinases can activate the transcription factor CREB, promoting high expression of key P450 genes in the gut that confer resistance to nicotine‐based insecticides. However, these kinases can also activate the transcription factor CncC, which may lead to abnormal ovarian development and associated reproductive costs (Fu *et al*., 2024). Therefore, we speculate that the higher expression of detoxification genes and hormone pathway key genes in response to adverse stress in the guts of YPM larvae may incur fitness costs thus negatively affecting their growth.

Pathogenic microorganism infections often induce the synthesis of plant secondary metabolites, promoting plant resistance to insects (Kalaivani *et al*., 2018; Liu *et al*., 2018). Therefore, we further investigated the changes in metabolites in Ta‐treated corn through metabolomic analysis. The results revealed significant alterations in numerous metabolites, leading to the identification of wogonin, a flavonoid secondary metabolite, which notably increased after corn infection. Subsequent feeding experiments under laboratory conditions demonstrated that consumption of artificial feed containing wogonin effectively suppressed the growth of YPM larvae and elevated the mortality rates. Furthermore, qPCR validation confirmed the upregulation of key genes (*Fdp*, *Ad1*, *Ad2*) in the insect juvenile hormone biosynthesis pathway, while the expression levels of genes (*JHeh*, *JHe*) in the hydrolysis pathway were inhibited, ultimately resulting in an accumulation of juvenile hormones. Additionally, two chitin synthesis enzyme genes associated with cuticle formation were significantly upregulated after 2 days of consuming wogonin‐contained artificial feed, whereas their expression levels markedly decreased after 4 and 6 days. Specialized metabolites constitute the most crucial and diverse component of a plant's defensive arsenal. Identification and functional analysis of plant‐specific secondary metabolites induced by herbivory or fungi have been reported in various host plants. For instance, root colonization by *Tricboderma* can also augment the direct plant defence response to herbivorous insects by modulating the production of plant secondary metabolites (e.g. phenylpropanoids, flavonoids, terpenoids, alkaloids and so on) (Coppola *et al*., 2019; Leilo *et al*., 2023). These secondary metabolites exhibit diversity in type and play distinct roles in plant defence against external stressors. Among these, flavonoids are widely distributed in the plant kingdom and play a pivotal role in plant resistance to insects (Onkokesung *et al*., 2014; Xia *et al*., 2021). For instance, sustained accumulation of the flavonoid compound quercetin‐3‐O‐glucoside in tealeaves induced by *Ectropis grisescens* larvae further impedes continuous larval feeding (Jing *et al*., 2024). Additionally, certain flavonoids can serve as phytoalexin, participating in plant–microorganism interactions. For example, sakuranetin identified in rice leaves infected with rice blast fungus exhibits diverse antifungal activities (Murata *et al*., 2020). Furthermore, studies have revealed that supplementing artificial feed with flavonoids can effectively inhibit the growth of phloem‐sucking insects (Chen *et al*., 2022). Therefore, combining the findings of our study, the accumulation of wogonin, a flavonoid compound induced by pathogenic microorganism infection in corn, may be critical in inhibiting the growth of YPM larvae.

The internal homeostasis of insect hormones plays a crucial role in alleviating the fitness cost generated during the adaptive evolution process of insects. The diamondback moth acquires high resistance to Bt protein by increasing the content of the moulting hormone 20E, while also maintaining homeostasis of 20E in the body through negative feedback regulation, enabling the diamondback moth to develop high resistance to Bt biopesticides while maintaining normal growth and development (Guo *et al*., 2024). In our study, the increased juvenile hormone content observed in YPM larvae fed on wogonin‐contained artificial diet may represent an adaptive defence strategy against abiotic stressors, with disrupted hormone homeostasis potentially contributing to fitness costs. Interestingly, the toxic effects of secondary metabolites extend beyond directly targeting insects. For example, studies have shown that the secondary metabolite sakuranetin in rice can inhibit beneficial endosymbiotic bacteria in the brown plant hopper, indirectly defending against herbivorous insects and revealing a novel plant defence mechanism (Liu *et al*., 2023). Similarly, we found that wogonin significantly inhibits the abundance of core gut bacteria in YPM larvae, suggesting that the imbalance between hormone regulation and microbial abundance may ultimately inhibit larval growth. However, the impact of wogonin on the regulation of juvenile hormone homeostasis in YPM and the role of its core gut bacterium, *Enterococcus*, require further investigation. Furthermore, the utilization of wogonin and the application of *Trichoderma*‐based biopesticides in field holds considerable value for the future management of YPM populations. Interestingly, wogonin exhibits diverse biological activities such as anti‐cancer, anti‐inflammation and treatment of bacterial and viral infections in the medical realm (Huynh *et al*., 2020). Thus, it is essential to consider how to optimize its application and potential in the management of field pests and diseases, as well as its implications for human health. Moreover, the exploration of a broader spectrum of insect‐resistant secondary metabolites in *Trichoderma*‐infected corn is crucial for enriching the database of insect‐resistance compounds, as well as for the identification of resistance genes and the breeding of pest‐resistant varieties.

## Conclusion

In summary, this study provides comprehensive evidence from the perspectives of both offspring and host plant defence, supporting the validity of parental insects' preference behaviour based on environmental experiences. Our findings confirm that the experience conclusion of ‘Mother Knows Worst’ is similarly applicable within the YPM‐microbe–plant interaction model. Furthermore, we elucidated the mechanisms by which pathogenic microorganisms mediate host plants resistance to insects, particularly through the induction of secondary metabolites like wogonin. These insights enhance our understanding of the intricate interactions among YPM, microbes and host plants, offering a theoretical foundation for the development of effective biological control strategies against YPM.

## Materials and methods

### Insect rearing and growth conditions

The YPM population used in this study was reared for over 100 generations on corn in a climatic incubator maintained at 25 ± 2 °C, under a photoperiod of 16 h light and 8 h dark, with a light intensity of 3500 lux. Eggs laid by female YPMs were collected within 48 h, selectively excluding darker‐coloured eggs that may indicate irregular oviposition times. These eggs were subsequently incubated in a climate‐controlled environment. Four‐day‐old larvae with consistent body length and size were selected for subsequent experiments.

### Corn treatments and insect bioassays

The preparation of *Trichoderma asperellum* spore suspension and corn treatments followed previous protocols (Chen *et al*., 2023). The detail of preparation was listed in Appendix S1.

To initially assess whether YPM larvae exhibit non‐selective feeding behaviour towards *T. asperellum*‐treated (hereinafter referred to as Ta‐treated) corn, we placed both Ta‐treated and healthy corn kernels in petri dishes for a selection experiment. Considering the small size and extremely soft cuticle of first‐instar larvae, we chose to use second‐instar larvae (approximately 4 days old) for subsequent experiments to avoid potential negative effects caused by mechanical damage during handling. Each trial involved 10 larvae, with the experiment repeated ten times. Subsequently, we transferred 10 larvae to each type of corn (Ta‐treated and healthy) and observed them over periods of 2, 4 and 6 days for a non‐selective test. Following incubation periods of 2, 4 and 6 days, the larvae will be picked from the corn to measure their weight and body length. Each treatment will consist of four biological replicates.

### Bacterial and transcriptome sequencing of YPM larvae gut

Intestinal tissues of YPM larvae fed on healthy and fungus‐infected corn for 2, 4 and 6 days were collected separately for DNA and RNA extraction. Before extraction, insects were starved for 2 h to reduce gut contamination from food residues. Sufficient larval gut tissue was then dissected and preserved in liquid nitrogen for subsequent extraction. DNA and RNA extraction followed the protocol outlined in our previous study (Chen *et al*., 2023). The bacterial composition of the YPM larvae gut was analysed by sequencing the *16S rRNA* gene. Meanwhile the mRNA enrichment of gut tissue was accomplished using Oligo (dT) magnetic beads, after which isolated mRNA was randomly fragmented with divalent cations in NEB Fragmentation Buffer to prepare a library for Illumina sequencing. The detail information of 16S rRNA and transcriptome sequencing method was listed in Appendix S1. The sequence data were deposited in the Figshare database (Li *et al*., 2024).

### Extensive targeted metabolomics analysis before and after *Trichoderma asperellum* inoculation in corn

To conduct extensive targeted metabolomic analysis, we collected sufficient quantities of Ta‐treated corn, healthy corn and *Trichoderma asperellum* spore suspension. After all samples were processed to obtain the supernatant, filtered it through a microporous membrane (0.22 μm pore size) and stored it in an injection vial for UPLC‐MS/MS analysis. The extracts were analysed using a UPLC‐ESI‐MS/MS system (UPLC, ExionLCTM AD) and tandem mass spectrometry (https://sciex.com.cn). The detail information of metabolomic analysis was listed in Appendix S1.

### Evaluation of the secondary metabolite wogonin on the growth of YPM larvae

The methods of body soaking and adding wogonin into artificial diet were applicated to assess the impact of wogonin on the growth of YPM larvae. For body soaking method, wogonin was dissolved in methanol to prepare solutions with concentrations of 1 mg/g, 2 mg/g and 4 mg/g. Second‐instar larvae were immersed in these solutions for 10 s before being transferred onto healthy corn for continued feeding. After 24 h, larval weight and mortality rate were measured, with 10 replications for each treatment. Additionally, wogonin was added to artificial diet at final concentrations of 1 mg/g, 2 mg/g and 4 mg/g for a 6‐day feeding period, followed by measurements of larval weight and mortality rate with 15 repetitions per treatment.

### Quantitative PCR and insect hormone determination

To validate the authenticity of differentially expressed genes across treatments, qRT‐PCR was conducted using specific primers listed in Table S1. The expression levels of target genes and core bacteria were normalized to the YPM housekeeping gene (Jia *et al*., 2016). Each RT‐qPCR reaction was performed in a 20.0 μL reaction mixture consisting of 10.0 μL of 2× SYBR Green PCR Master Mix, 0.4 μL of each primer, 2.0 μL of cDNA sample (at a concentration of 100 ng/μL) and 7.2 μL of sterilized ultrapure H_2_O. The cycling conditions were set as follows: an initial denaturation at 95 °C for 3 min, followed by 40 cycles of denaturation at 95 °C for 10 s and annealing/extension at 60 °C for 30 s to record the dissociation curves. Each experiment included blank controls containing sterilized ultrapure H_2_O in place of the template. Each treatment included three biological replicates, with each replicate comprising three technical replicates.

For the treated groups, samples were collected and transferred into electropolished tubes. Following the addition of 4 mL PBS solution, the YPM larvae were homogenized and aliquoted into two 1.5 mL tubes. Supernatants were obtained by centrifugation at 5000 r/min at 4 °C and analysed for Juvenile III levels using an ELISA kit (JM‐1520004102, JINGMEI, Inc., China). Absorbance was measured at 450 nm using a blank well as the control. Sample concentrations of Juvenile III were derived from the standard curve using linear regression based on standard concentrations and OD values. Each test included three biological replicates.

### Statistical analysis

Treatment effects were assessed with analysis of variance (ANOVA) or Student's *t*‐test using IBM SPSS statistics version 21. For assays in which two or more treatments were compared, Tukey's honest significant difference (HSD) multiple comparison test (*P* < 0.05) was used to determine whether the treatments were significantly different.

## Author contributions

QL and YLD conceived and designed the experiments. JYL and YT performed the experiments. KNW and AHZ analysed the data. QL wrote the manuscript. All the authors read and approved the final version of the manuscript.

## Conflicts of interest

The authors declare that no conflict of interest exists.

## Supporting information


**Appendix S1** Methods.
**Figure S1** Comparative analysis of detoxification enzyme activity in YPM larvae after feeding on *T. asperellum* infected corn at different time.
**Figure S2** Functional annotation of the gene set associated with pink modules.
**Figure S3** Composition and classification of differential metabolites in *T. asperellum* infected corn and healthy corn.
**Figure S4** KEGG classification analysis of differential metabolites in *T.asperellum* infected corn and healthy corn.
**Figure S5** KEGG enrichment analysis of differential metabolites in *T.asperellum* infected corn and healthy corn.

**Table S1** Metabolomic analysis of *T. asperellum*‐infected corn, healthy corn and *T. asperellum*.

## Data Availability

The data that support the findings of this study are openly available in Figureshare at https://doi.org/10.6084/m9.figshare.27908574 (Li *et al*., 2024).

## References

[pbi70051-bib-0001] Becher, P.G. , Flick, G. , Rozp dowska, E. , Rozpędowska, E. , Schmidt, A. , Hagman, A. , Lebreton, S. *et al*. (2012) Yeast, not fruit volatiles mediate *Drosophila melanogaster* attraction, oviposition and development. Funct. Ecol. 26(4), 822–828.

[pbi70051-bib-0002] Cha, D.H. , Hesler, S.P. , Brind'Amour, G. , Wentworth, K.S. , Villani, S. , Cox, K.D. , Boucher, M.T. *et al*. (2020) Behavioral evidence for contextual olfactory‐mediated avoidance of the ubiquitous phytopathogen *Botrytis cinerea* by *Drosophila suzukii* . Insect Sci. 27(4), 771–779. 31087762 10.1111/1744-7917.12691

[pbi70051-bib-0003] Chen, Q.Q. , He, J. , Ma, C. , Yu, D. and Kang, L. (2015) Syntaxin 1A modulates the sexual maturity rate and progeny egg size related to phase changes in locusts. Insect Biochem. Mol. Biol. 56, 1–8. 25446392 10.1016/j.ibmb.2014.11.001

[pbi70051-bib-0004] Chen, S. , Sun, B. , Shi, Z.Y. , Miao, X. and Li, H. (2022) Identification of the rice genes and metabolites involved in dual resistance against brown planthopper and rice blast fungus. Plant Cell Environ. 45, 1914–1929. 35343596 10.1111/pce.14321

[pbi70051-bib-0005] Chen, Y. , Han, J. , Yang, H. , Qin, X. , Guo, H. and du, Y. (2023) Different maize ear rot fungi deter the oviposition of yellow peach moth (*Conogethes punctiferalis* (Guenée)) by maize volatile organic compounds. Agronomy 13, 251.

[pbi70051-bib-0006] Coppola, M. , Cascone, P. , Lelio, I. , Di Lelio, I. , Woo, S.L. , Lorito, M. , Rao, R. *et al*. (2019) Trichoderma atroviride P1 colonization of tomato plants enhances both direct and indirect defense barriers against insects. Front. Physiol. 10, 3389. 10.3389/fphys.2019.00813 PMC6624734 31333483

[pbi70051-bib-0007] Eberl, F. , Uhe, C. and Unsicker, S.B. (2019) Friend or foe. The role of leaf‐inhabiting fungal pathogens and endophytes in tree‐insect interactions. Fungal Ecol. 38, 104–112.

[pbi70051-bib-0008] Eberl, F. , Bobadilla, M.F.D. , Reichelt, M. , de Fernanz Bobadilla, M. , Hammerbacher, A. , Gershenzon, J. and Unsicker, S.B. (2020) Herbivory meets fungivory: Insect herbivores feed on plant pathogenic fungi for their own benefit. Ecol. Lett. 23(7), 1073–1084. 32307873 10.1111/ele.13506

[pbi70051-bib-0009] Franco, F.P. , Túler, A.C. , Gallan, D.Z. , Gonçalves, F.G. , Favaris, A.P. , Peñaflor, M.F.G.V. , Leal, W.S. *et al*. (2021) Fungal phytopathogen modulates plant and insect responses to promote its dissemination. ISME J. 15(12), 3522–3533. 34127802 10.1038/s41396-021-01010-z PMC8630062

[pbi70051-bib-0010] Fu, B.L. , Liang, J.J. , Hu, J.Y. , du, T. , Tan, Q. , He, C. , Wei, X. *et al*. (2024) GPCR–MAPK signaling pathways underpin fitness trade‐offs in whitefly. Proc. Natl. Acad. Sci. 121, e2402407121. 38959045 10.1073/pnas.2402407121 PMC11252912

[pbi70051-bib-0011] Gao, M.J. , He, Y. , Yin, X. , Gao, M. , Zhong, X. , Yan, B. , Wu, Y. *et al*. (2021) Ca^2+^ sensor‐mediated ROS scavenging suppresses rice immunity and is exploited by a fungal effector. Cell 184(21), 5391–5404. 34597584 10.1016/j.cell.2021.09.009

[pbi70051-bib-0012] Gould, F. , Brown, Z.S. and Kuzma, J. (2018) Wicked evolution: Can we address the sociobiological dilemma of pesticide resistance? Science 360, 728–732. 29773742 10.1126/science.aar3780

[pbi70051-bib-0013] Gu, F. , Ai, S.P. , Chen, Y.Y. , Jin, S. , Xie, X. , Zhang, T. , Zhong, G. *et al*. (2022) Mutualism promotes insect fitness by fungal nutrient compensation and facilitates fungus propagation by mediating insect oviposition preference. ISME J. 16, 1831–1842. 35418221 10.1038/s41396-022-01237-4 PMC9213550

[pbi70051-bib-0014] Guo, H.G. , Han, C.Y. , Zhang, A.H. , Yang, A.Z. , Qin, X.C. , Zhang, M.Z. and du, Y.L. (2022) Penicillium fungi mediate behavioral responses of the yellow peach moth, *Conogethes punctiferalis* (Guenée) to apple fruits via altering the emissions of host plant VOCs. Arch. Insect Biochem. Physiol. 110, e21895. 35373383 10.1002/arch.21895

[pbi70051-bib-0015] Guo, Z.J. , Zhu, L.H. , Cheng, Z.Q. , Dong, L. , Guo, L. , Bai, Y. , Wu, Q. *et al*. (2024) A midgut transcriptional regulatory loop favors an insect host to withstand a bacterial pathogen. Innovations 5, 100675. 10.1016/j.xinn.2024.100675 PMC11338098 39170942

[pbi70051-bib-0016] He, J. , Zhu, Y.N. , Wang, B.C. , Wang, B. , Yang, P. , Guo, W. , Liang, B. *et al*. (2022) piRNA‐guided intron removal from pre‐mRNAs regulates density‐dependent reproductive strategy. Cell Rep. 39(4), 110593. 35476998 10.1016/j.celrep.2022.110593

[pbi70051-bib-0017] Heard, E. and Martienssen, R.A. (2014) Transgenerational epigenetic inheritance: myths and mechanisms. Cell 157, 95–109. 24679529 10.1016/j.cell.2014.02.045 PMC4020004

[pbi70051-bib-0018] Hu, X.Y. , Su, S.L. , Liu, Q.S. , Hu, X. , Su, S. , Liu, Q. , Jiao, Y. *et al*. (2020) Caterpillar‐induced rice volatiles provide enemy‐free space for the offspring of the brown planthopper. elife 9, e55421. 32778222 10.7554/eLife.55421 PMC7419140

[pbi70051-bib-0019] Huynh, D.L. , Ngau, T.H. , Nguyen, N.H. , Tran, G.B. and Nguyen, C.T. (2020) Potential therapeutic and pharmacological effects of Wogonin: an updated review. Mol. Biol. Rep. 47, 9779–9789. 33165817 10.1007/s11033-020-05972-9

[pbi70051-bib-0020] Jia, X.J. , Wang, H.X. , Yan, Z.G. , Zhang, M.Z. , Wei, C.H. , Qin, X.C. , Ji, W.R. *et al*. (2016) Antennal transcriptome and differential expression of olfactory genes in the yellow peach moth, *Conogethes punctiferalis* (Lepidoptera: Crambidae). Sci. Rep. 6, 29067. 27364081 10.1038/srep29067 PMC4929561

[pbi70051-bib-0021] Jiao, Y.Y. , Hu, X.Y. , Peng, Y.F. , Jiao, Y. , Hu, X. , Peng, Y. , Wu, K. *et al*. (2018) Bt rice plants may protect neighbouring non‐Bt rice plants against the striped stem borer, *Chilo suppressalis* . Proc. R. Soc. B 285(1883), 20181283. 10.1098/rspb.2018.1283 PMC6083243 30051874

[pbi70051-bib-0022] Jing, T.T. , Du, W.K. , Qian, X.N. , Jing, T. , Du, W. , Qian, X. , Wang, K. *et al*. (2024) UGT89AC1‐mediated quercetin glucosylation is induced upon herbivore damage and enhances *Camellia sinensis* resistance to insect feeding. Plant Cell Environ. 47(2), 682–697. 37882446 10.1111/pce.14751

[pbi70051-bib-0023] Juottonen, H. , Moghadam, N.N. , Murphy, L. , Mappes, J. and Galarza, J.A. (2022) Host's genetic background determines the outcome of reciprocal faecal transplantation on life‐history traits and microbiome composition. Anim. Microbiome 4, 67. 36564793 10.1186/s42523-022-00210-y PMC9789590

[pbi70051-bib-0024] Kalaivani, K. , Kalaiselvi, M.M. and Senthil‐Nathan, S. (2018) Effect of Methyl Salicylate (MeSA) induced changes in rice plant (*Oryza sativa*) that affect growth and development of the rice leaffolder, *Cnaphalocrocis medinalis* . Physiol. Mol. Plant Pathol. 101, 116–126.

[pbi70051-bib-0025] Keesey, I.W. , Koerte, S. , Khallaf, M.A. , Retzke, T. , Guillou, A. , Grosse‐Wilde, E. , Buchon, N. *et al*. (2017) Pathogenic bacteria enhance dispersal through alteration of Drosophila social communication. Nat. Commun. 8, 265. 28814724 10.1038/s41467-017-00334-9 PMC5559524

[pbi70051-bib-0026] Kohandani, F. , Goff, G.J.L. and Hance, T. (2017) Does insect mother know under what conditions it will make their offspring live? Insect Sci. 24, 141–149. 26616755 10.1111/1744-7917.12300

[pbi70051-bib-0027] Leilo, I.D. , Forni, G. , Magoga, G. , Lelio, D.I. , Brunetti, M. , Bruno, D. , Becchimanzi, A. *et al*. (2023) A soil fungus confers plant resistance against a phytophagous insect by disrupting the symbiotic role of its gut microbiota. Proc. Natl. Acad. Sci. USA 120(10), e2216922120. 36848561 10.1073/pnas.2216922120 PMC10013743

[pbi70051-bib-0028] Li, Q. , Li, W.Y. , Jin, Z.Y. , Li, W. , Jin, Z. , Li, J. , Xue, D. *et al*. (2024) Penicillium‐infected apples benefit larval development of *Conogethes punctiferalis* via alterations of their gut bacteria community and gene expression. J. Agric. Food Chem. 72(14), 7774–7783. 38563445 10.1021/acs.jafc.3c09614

[pbi70051-bib-0029] Li, J.Y. , Ni, B.Q. , Wu, Y.N. , Yang, Y. , Mu, D. , Wu, K.N. , Zhang, A. *et al*. (2024) The cultivable gut bacteria *Enterococcus mundtii* promotes early‐instar larval growth of *Conogethes punctiferalis* via enhancing digestive enzyme activity. Pest Manag. Sci. 80, 6179–6188. 39072862 10.1002/ps.8346

[pbi70051-bib-0030] Li, Q. , Li, J.Y. , Wu, K.N. , Tong, Y. , Zhang, A.H. and Du, Y.L. (2024) Related data of 16S rRNA and transcriptome sequencing of yellow peach moth. Figshare. 10.6084/m9.figshare.27908574

[pbi70051-bib-0031] Lin, Y. , Lin, S. , Akutse, K.S. , Hussain, M. and Wang, L. (2016) Diaphorina citri Induces Huanglongbing‐infected citrus plant volatiles to repel and reduce the performance of *Propylaea japonica* . Front. Plant Sci. 7, 1969. 28083006 10.3389/fpls.2016.01969 PMC5183590

[pbi70051-bib-0032] Liu, X.L. , Li, J.C. , Xu, L.P. , Wang, Q. and Lou, Y. (2018) Expressing OsMPK4 impairs plant growth but enhances the resistance of rice to the striped stem borer *Chilo suppressalis* . Int. J. Mol. Sci. 19, 1. 10.3390/ijms19041182 PMC5979284 29652796

[pbi70051-bib-0033] Liu, M.Y. , Hong, G.J. , Li, H.J. , Liu, M. , Hong, G. , Li, H. , Bing, X. *et al*. (2023) Sakuranetin protects rice from brown planthopper attack by depleting its beneficial endosymbionts. Proc. Natl. Acad. Sci. USA 120(23), e2305007120. 37256931 10.1073/pnas.2305007120 PMC10266023

[pbi70051-bib-0034] Ma, M.Q. , Luo, J. , Chen, X.T. , Li, C. , Li, S. , Sun, J. and Xu, L. (2024) Gut bacteria facilitate leaf beetles in adapting to dietary specialization by enhancing larval fitness. NPJ Biofilms. Microbiomes 10, 110. 39438487 10.1038/s41522-024-00587-5 PMC11496516

[pbi70051-bib-0035] Mazumdar, T. , Teh, B.S. , Murali, A. , Schmidt‐Heck, W. , Schlenker, Y. , Vogel, H. and Boland, W. (2021) Transcriptomics reveal the survival strategies of *Enterococcus mundtii* in the gut of *Spodoptera littoralis* . J. Chem. Ecol. 47, 227–241. 33459999 10.1007/s10886-021-01246-1

[pbi70051-bib-0036] Murata, K. , Kitano, T. , Yoshimoto, R. , Takata, R. , Ube, N. , Ueno, K. , Ueno, M. *et al*. (2020) Natural variation in the expression and catalytic activity of a naringenin 7‐O‐methyltransferase influences antifungal defenses in diverse rice cultivars. Plant J. 101, 1103–1117. 31630460 10.1111/tpj.14577

[pbi70051-bib-0037] Onkokesung, N. , Reichelt, M. , van Doorn, A. , Schuurink, R.C. , van Loon, J.J.A. and Dicke, M. (2014) Modulation of flavonoid metabolites in Arabidopsis thaliana through overexpression of the MYB75 transcription factor: role of kaempferol‐3,7‐dirhamnoside in resistance to the specialist insect herbivore *Pieris brassicae* . J. Exp. Bot. 65(8), 2203–2217. 24619996 10.1093/jxb/eru096 PMC3991749

[pbi70051-bib-0038] Raman, A. and Suryanarayanan, T.S. (2017) Fungus‐plant interaction influences plant‐feeding insects. Fungal Ecol. 29, 123–132.

[pbi70051-bib-0039] Shao, Y.Q. , Mason, C.J. and Felton, G.W. (2024) Toward an integrated understanding of the lepidoptera microbiome. Annu. Rev. Entomol. 69, 117–137. 37585608 10.1146/annurev-ento-020723-102548

[pbi70051-bib-0040] Stensmyr, M.C. , Dweck, H.K. , Farhan, A. , Ibba, I. , Strutz, A. , Mukunda, L. , Linz, J. *et al*. (2012) A conserved dedicated olfactory circuit for detecting harmful microbes in Drosophila. Cell 151(6), 1345–1357. 23217715 10.1016/j.cell.2012.09.046

[pbi70051-bib-0041] Xia, J.X. , Guo, Z.J. , Yang, Z.Z. , Xia, J. , Guo, Z. , Yang, Z. , Han, H. *et al*. (2021) Whitefly hijacks a plant detoxification gene that neutralizes plant toxins. Cell 184(7), 1693–1705. 33770502 10.1016/j.cell.2021.02.014

[pbi70051-bib-0042] Zhu, Y.N. , He, J. , Wang, J.W. , Guo, W. , Liu, H. , Song, Z. and Kang, L. (2024) Parental experiences orchestrate locust egg hatching synchrony by regulating nuclear export of precursor miRNA. Nat. Commun. 15(1), 4328. 38773155 10.1038/s41467-024-48658-7 PMC11109280

