# Supplementary material for: Mother knows worst? Fungal infection enhances corn flavonoid of wogonin to inhibit Conogethes punctiferalis larval growth
Source: Plant Biotechnol J. 2025 Mar 19;23(6):2254–65. doi: 10.1111/pbi.70051 (PMC12120905; doi:10.1111/pbi.70051)
Supplement: Supplementary file 1 — Appendix S1 Methods. Figure S1 Comparative analysis of detoxification enzyme activity in YPM larvae after feeding on T. asperellum infected corn at different time. Figure S2 Functional annotation of the gene set associated with pink modules. Figure S3 Composition and classification of differential metabolites in T. asperellum infected corn and healthy corn. Figure S4 KEGG classification analysis of differential metabolites in T.asperellum infected corn and healthy corn. Figure S5 KEGG enrichment analysis of differential metabolites in T.asperellum infected corn and healthy corn. [file PBI-23-2254-s001.docx]

**Mother Knows Worst? Fungal infection enhances corn flavonoid of wogonin to inhibit Conogethes punctiferalis larval growth**

Qian Li^1^, Jiayu Li^1^, Kaining Wu^1^, Yue Tong^1^, Aihuan Zhang^1^, Yanli Du^1*^

1 College of Bioscience and Resource Environment/Key Laboratory of Urban Agriculture (North China), Ministry of Agriculture and Rural Affairs of the People’s Republic of China, Beijing University of Agriculture, Beijing, China

^*^Corresponding author: Yanli Du (yanlidu@126.com)

**Supplementary Methods**

**Corn infection treatment**

Conidia from 7‐day‐old *T. asperellum* cultures grown on potato‐dextrose agar were gently washed with 1.5 mL of sterilized distilled water, and the density of the conidial suspension was adjusted to 8 × 10^8^ conidia/ mL. Corn with uniform size and shape were purchased from the supermarket at Beijing University of Agriculture and stored at 4°C for subsequent experiments. Corn segments weighing 80 g each were longitudinally slit, and 125 μL of the microbial suspension was injected into each row, totaling 2 mL per segment. These treated segments were designated as Ta-treated corn, while healthy corn segments injected with sterile water served as the control group. After injection, incubate the segments for 72 hours before introducing YPM larvae. Eggs from the same YPM batch were collected, and first-instar larvae hatched within 24 hours were selected for individual rearing. To avoid mechanical damage due to their small size, larvae were reared for four days before being used in subsequent experiments.

**Bacterial and transcriptome sequencing of YPM larvae gut**

The bacterial composition of the YPM larvae gut was analyzed by sequencing the *16S rRNA* gene. The V3-V4 region of the bacterial *16S rRNA* gene was amplified using primers 341F (5’-CCTAYGGGRBGCASCAG-3’) and 806R (5’-GGACTACNNGGGTATCTAAT-3’) [1]. To ensure statistical reliability and biological validity, we implemented strict quality controls throughout the data processing steps, including read utilization, tag assembly, and OTU (Operational Taxonomic Units) clustering. Post-sequencing, low-quality reads were filtered out, paired-end reads were assembled into tags, and these tags were further refined to generate clean tags. The clean data were clustered into OTUs at a 97% sequence identity threshold using the UPARSE (v7.0) OTU algorithm [2]. Functional predictions of the YPM microbiota were made using *16S rRNA* data and the Phylogenetic Investigation of Communities by Reconstruction of Unobserved States (PICRUSt), based on the Green Genes database [3].

Given the critical role of the gut system in insect metabolic functions, we focused on the gut to elucidate differential gene expression changes in response to feeding. Using gut transcriptome sequencing, we investigated differentially expressed genes in the YPM larval gut when fed Ta-treated corn versus healthy corn. Total RNA was extracted from the guts of YPM larvae fed on both types of corn at various time points using a total RNA extraction reagent kit (Tianmo, Beijing, China), following the manufacturer’s instructions. RNA quality was assessed via 1% (w/v) agarose gel electrophoresis and quantified with a Nanodrop 20000 spectrophotometer (DNovix, Washington, DC, USA). mRNA enrichment was accomplished using Oligo (dT) magnetic beads, after which isolated mRNA was randomly fragmented with divalent cations in NEB Fragmentation Buffer to prepare a library for Illumina sequencing. Due to the lack of genomic information for YPM, we employed Trinity software for de novo assembly of the reads, followed by differential expression analysis of the genes (DEGs).

**Extensive targeted metabolomics analysis before and after *Trichoderma asperellum* inoculation in corn**

Using vacuum freeze-drying technology, we lyophilized the biological samples in a lyophilizer (Scientz-100F) and then ground them into a powder using a grinder (MM 400, Retsch) at 30 Hz for 1.5 minutes. Next, we weighed out 50 mg of the powdered sample with an electronic balance (MS105DΜ) and added 1200 μL of pre-cooled (-20 °C) 70% methanolic aqueous internal standard extract (adding less than 50 mg of sample at a ratio of 1200 μL extractant per 50 mg sample). The mixture was vortexed for 30 seconds every 30 minutes, repeated six times. Following centrifugation at 12,000 rpm for 3 minutes, we aspirated the supernatant, filtered it through a microporous membrane (0.22 μm pore size), and stored it in an injection vial for UPLC-MS/MS analysis. The extracts were analyzed using a UPLC-ESI-MS/MS system (UPLC, ExionLCTM AD) and tandem mass spectrometry (<https://sciex.com.cn/>). The analytical conditions included an Agilent SB-C18 column (1.8 μm, 2.1 mm × 100 mm) with a mobile phase composed of solvent A (pure water with 0.1% formic acid) and solvent B (acetonitrile with 0.1% formic acid). Sample measurements employed a gradient program starting at 95% A and 5% B, transitioning linearly to 5% A and 95% B over 9 minutes, maintaining this composition for 1 minute, followed by a return to 95% A and 5% B over 1.1 minutes, and sustaining this for 2.9 minutes. The flow rate was set at 0.35 mL/min, the column oven temperature at 40 °C, and the injection volume at 2 μL. The effluent was connected alternately to an ESI-triple quadrupole-linear ion trap (QTRAP)-MS. ESI source parameters included a source temperature of 500 °C, ion spray voltage of 5500 V (positive ion mode) or -4500 V (negative ion mode), and gas settings (GSI, GSII, CUR) at 50, 60, and 25 psi, respectively, with high collision-activated dissociation (CAD). QQQ scans were performed as MRM experiments with medium collision gas (nitrogen), and DP (declustering potential) and CE (collision energy) were optimized for individual MRM transitions. Specific MRM transitions were monitored based on metabolites eluted during each period.

Unsupervised PCA was conducted using the prcomp function within R ([www.r-project.org](http://www.r-project.org)), with data scaled to unit variance prior to analysis. Hierarchical cluster analysis (HCA) results for samples and metabolites were presented as heatmaps with dendrograms, while Pearson correlation coefficients (PCC) between samples were calculated using the cor-function in R and displayed as heatmaps only. Both HCA and PCC analyses were performed using the ComplexHeatmap package in R, visualizing normalized signal intensities of metabolites as a color spectrum.

For differential metabolite analysis between two groups, metabolites were determined by VIP (VIP > 1) and absolute Log_2_FC (|Log_2_FC| ≥ 1.0). VIP values were extracted from the OPLS-DA results, which also included score plots and permutation plots generated using the R package MetaboAnalystR. Data underwent log transformation (log2) and mean centering before OPLS-DA, and a permutation test (200 permutations) was conducted to avoid overfitting. Identified metabolites were annotated using the KEGG Compound database (<http://www.kegg.jp/kegg/compound/>) and subsequently mapped to the KEGG Pathway database (<http://www.kegg.jp/kegg/pathway.html>). Pathways with significantly regulated metabolites were subjected to metabolite set enrichment analysis (MSEA), with significance assessed via hypergeometric test p-values.

References

1. Hugerth LW, Wefer HA, Lundin S et al. DegePrime, a program for degenerate primer design for broad-taxonomic-range PCR in microbial ecology studies. Appl Environ Microbiol 2014; **80**: 5116-5123. doi: https://doi.org/10.1128/aem.01403-14.
2. Edgar RC. UPARSE: highly accurate OTU sequences from microbial amplicon reads. Nat Methods 2013; **10**: 996-998. doi: https://doi.org/10.1038/nmeth.2604.
3. Langille MGI, Zaneveld J, Caporaso JG et al. Predictive functional profiling of microbial communities using *16S rRNA* marker gene sequences. Nat Biotechnol 2013; **31**:814-821. doi: https://doi.org/10.1038/nbt.2676.


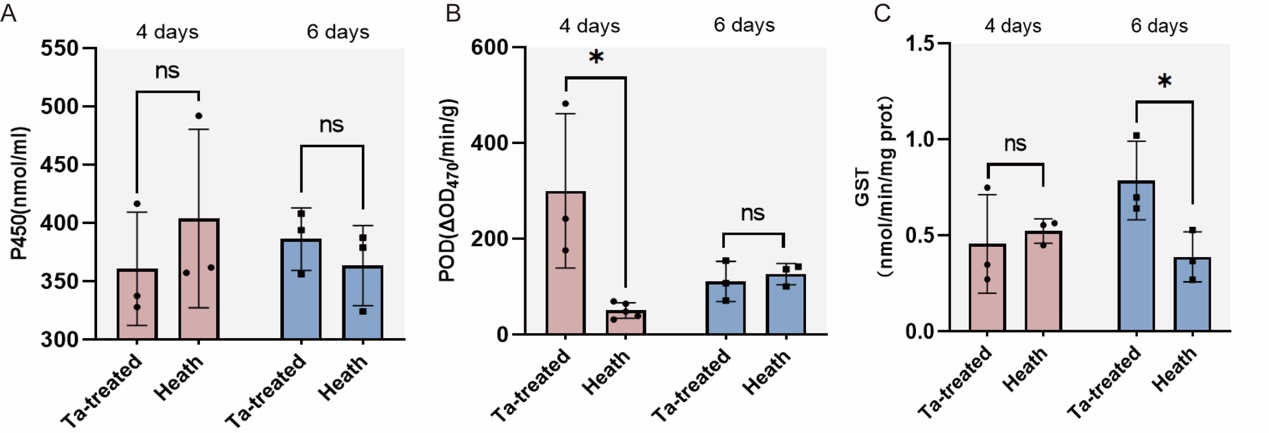


Figure S1. Comparative analysis of detoxification enzyme activity in YPM larvae after feeding on *T.asperellum* infected corn at different time. A. Cytochrome P450 enzyme activity in YPM larvae; B. Peroxidase enzyme activity in YPM larvae; C. Glutathione transferase enzyme activity in YPM larvae.


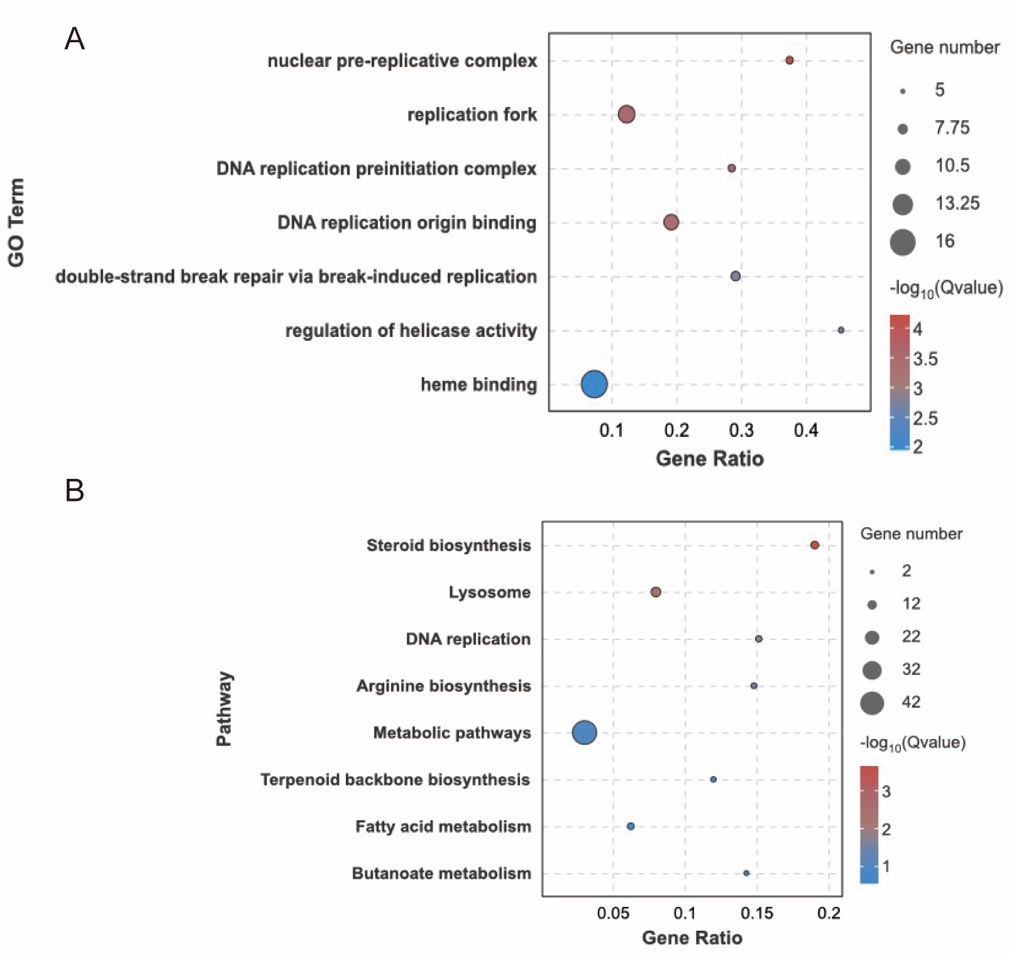


Figure S2. Functional annotation of the gene set associated with pink modules. A. GO functional analysis of gene sets associated with identified modules; B. KEGG functional analysis of gene sets associated with identified modules.


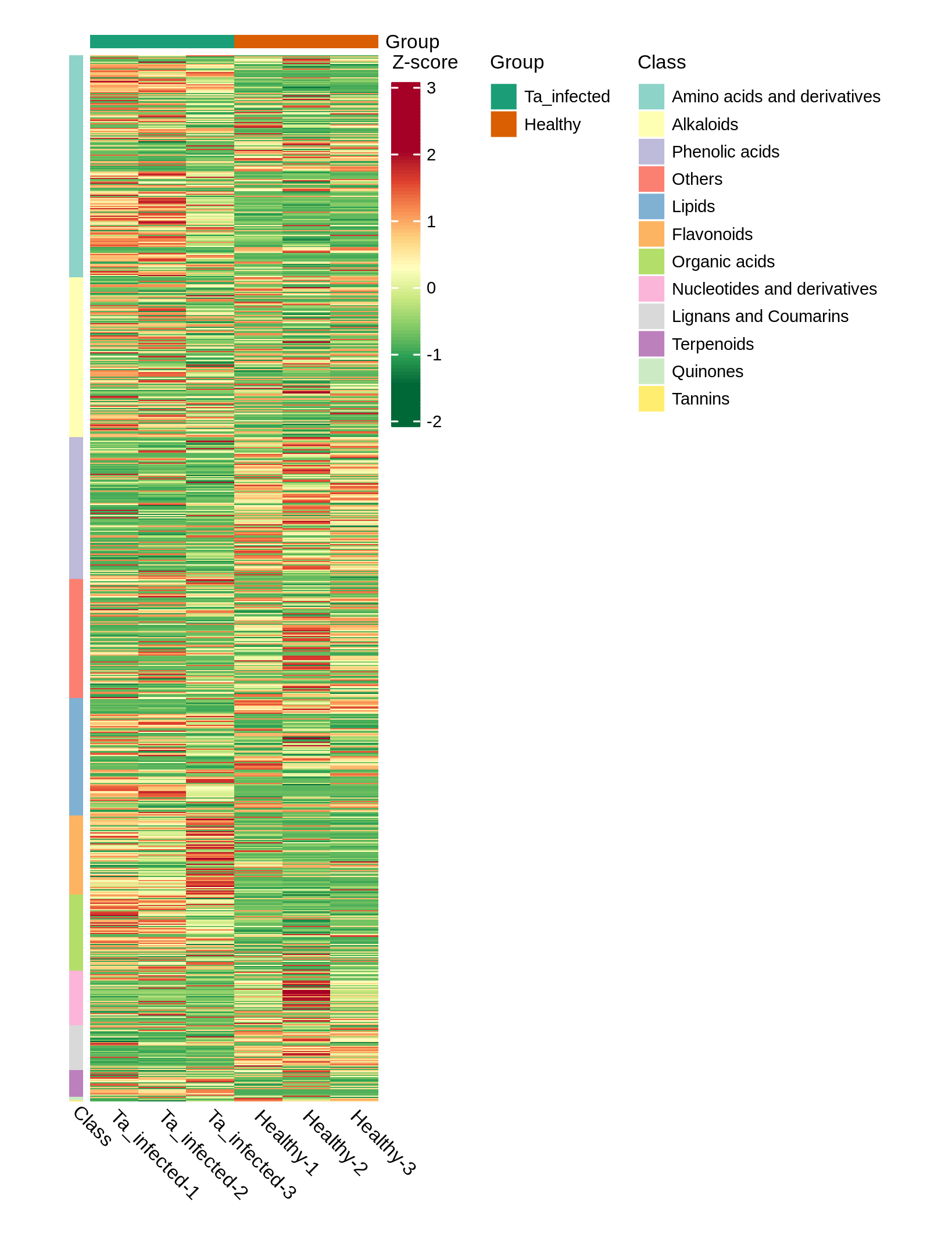


Figure S3. Composition and classification of differential metabolites in *T.asperellum* infected corn and healthy corn.


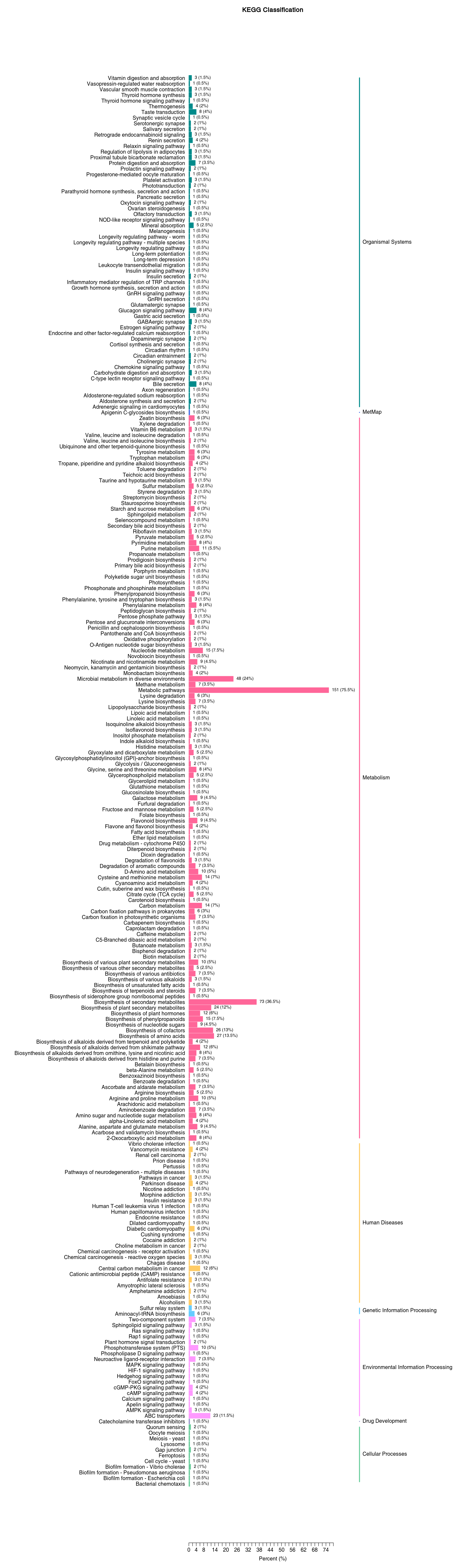


Figure S4. KEGG classification analysis of differential metabolites in *T.asperellum* infected corn and healthy corn.


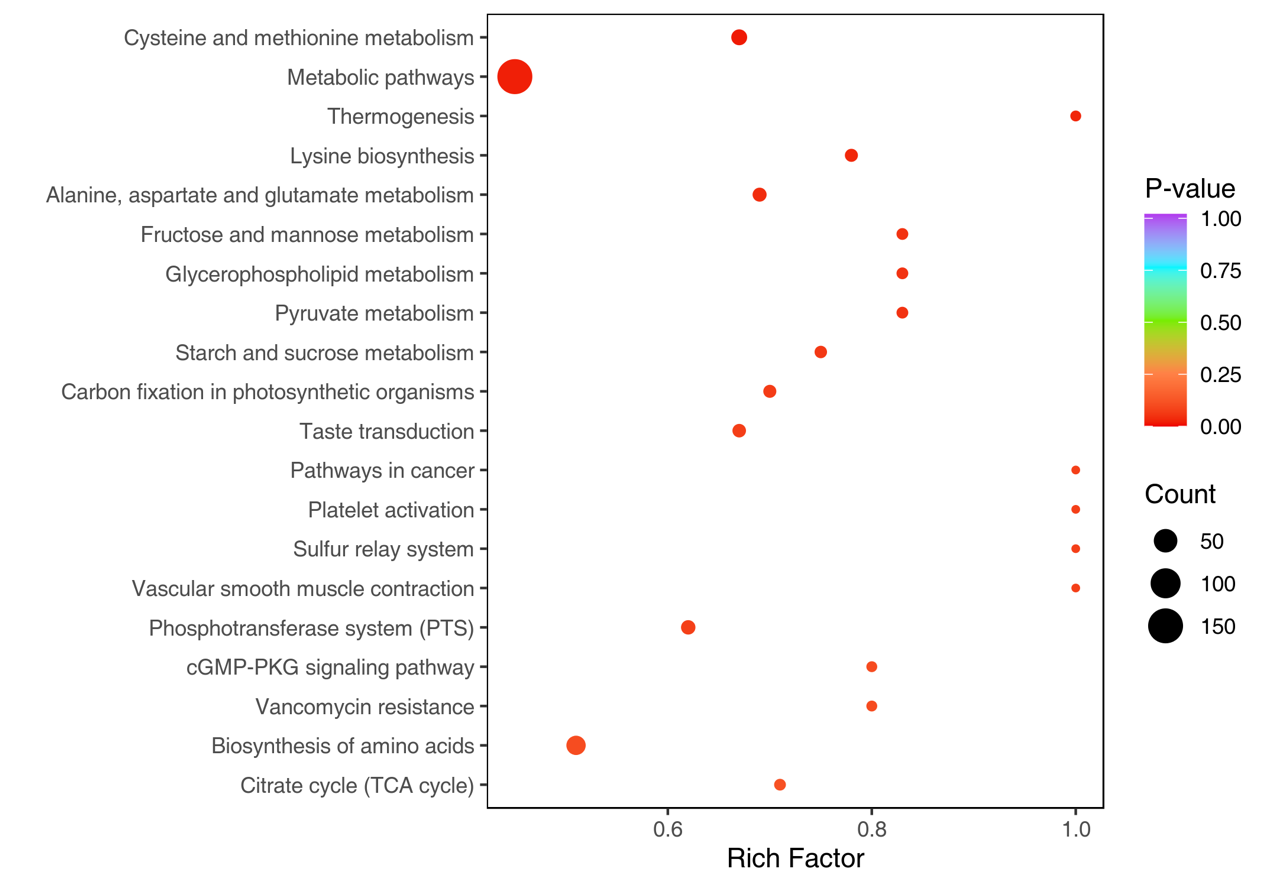


Figure S5. KEGG enrichment analysis of differential metabolites in *T.asperellum* infected corn and healthy corn.
